# Impact of Isoniazid Resistance-Conferring Mutations on the Clinical Presentation of Isoniazid Monoresistant Tuberculosis

**DOI:** 10.1371/journal.pone.0037956

**Published:** 2012-05-23

**Authors:** Raymund Dantes, John Metcalfe, Elizabeth Kim, Midori Kato-Maeda, Philip C. Hopewell, Masae Kawamura, Payam Nahid, Adithya Cattamanchi

**Affiliations:** 1 Division of Pulmonary and Critical Care Medicine, San Francisco General Hospital, University of California San Francisco, San Francisco, California, United States of America; 2 Curry International Tuberculosis Center, San Francisco General Hospital, University of California San Francisco, San Francisco, California, United States of America; 3 Tuberculosis Control Section, Department of Public Health, San Francisco, California, United States of America; St. Petersburg Pasteur Institute, Russian Federation

## Abstract

**Background:**

Specific isoniazid (INH) resistance conferring mutations have been shown to impact the likelihood of tuberculosis (TB) transmission. However, their role in the clinical presentation and outcomes of TB has not been evaluated.

**Methods:**

We included all cases of culture-confirmed, INH monoresistant tuberculosis reported to the San Francisco Department of Public Health Tuberculosis Control Section from October 1992 through October 2005. For cases with stored culture isolates, we used polymerase chain reaction (PCR) testing and gene sequencing to identify INH resistance-conferring mutations, and compared genotypic and phenotypic characteristics.

**Results:**

Among 101 consecutive cases of INH monoresistant TB in San Francisco 19 (19%) had isolates with a *katG* mutation other than S315T; 38 (38%) had isolates with the *katG* S315T mutation, 29 (29%) had isolates with a *inhA-15;c-t* promoter mutation, and 15 (15%) had isolates with other mutations. The *katG* S315T mutation was independently associated with high-level INH resistance (risk ratio [RR] 1.56, 95% confidence interval [CI] 1.07–2.27), and the *inhA-15;c-t* promoter mutation was inversely associated with high-level INH resistance (RR 0.43, 95% CI 0.21–0.89). However, specific INH resistance-conferring mutations were not associated with the clinical severity or outcomes of INH monoresistant TB cases.

**Conclusion:**

These data suggest that INH resistance-conferring mutations do not impact the clinical presentation of TB.

## Introduction

Resistance to first-line anti-tuberculosis (TB) medications is a continuing global health problem [1]. In particular, resistance to isoniazid (INH) is very common, with a prevalence of 10% among new cases and 28% among previously treated cases reported globally in 2009 [2]. Resistance to INH in *Mycobacterium tuberculosis* is mediated by at least two genes, *katG* [3] and *inh*A [4]. Mutations in other genes such as *ahpC* have been evaluated previously, and are considered compensatory mutations [5] that may be a marker for INH resistance, but do not appear to confer INH resistance [6]. Prior studies have shown that clinical outcomes are similar in patients with fully drug susceptible and INH monoresistant TB [7], and suggest that specific INH resistance-conferring mutations impact the transmissibility and pathogenicity of *M. tuberculosis* [8] [9]. Specifically, strains with the *katG* S315T mutation were associated with increased transmission and pathogenicity (measured by the proportion of cases that were considered as part of a chain of transmission) compared to INH-resistant strains with other mutations.

Because specific INH resistance-conferring mutations affect the ability of a strain to cause secondary cases, we hypothesized that they may also affect the host response and outcomes of infection. To test this hypothesis, we evaluated the genotypic and phenotypic characteristics of a well-characterized cohort of INH monoresistant TB cases in San Francisco.

## Methods

### Ethics statement

This study was approved by the University of California, San Francisco Human Research Protection Program. The requirement for informed consent was waived because this was a retrospective study and posed minimal risks to human subjects.

### Study population and setting

As previously described [7], we retrospectively abstracted data on demographic and clinical characteristics, treatment, and clinical outcomes for all cases of de novo INH monoresistant TB reported in San Francisco, California between October 1992 and November 2005. We excluded cases from this analysis if culture isolates were unavailable, known INH resistance-conferring genes were not successfully amplified, or the phenotypic level of INH-resistance was not known. In addition, we included only the first case from any group of clustered cases. Cases were considered to be clustered if their isolates demonstrated the same restriction fragment length polymorphism (RFLP) genotype based on the IS*6110* marker, the same drug resistance profile, the same drug resistance-conferring mutation, and belonged to the same *M. tuberculosis* lineage, as previously described [8]. Results presented below were not significantly different when all cases in a cluster were included in the analysis.

### Lineage

The three predominant phylogenetic lineages of *M. tuberculosis* in San Francisco are the East-Asian lineage, the Euro-American lineage, and the Indo-Oceanic lineage [10]. Multiplex real-time PCR was used to amplify lineage-specific markers [10], [11], [12], and each lineage was defined based on previously described methods [8].

### Drug resistance associated mutations

We identified INH resistance-conferring mutations from *M. tuberculosis* culture isolates stored at −80 degrees Celsius. Briefly, for all isolates with phenotypic resistance to INH we performed real time polymerase chain reaction (PCR) to identify the *katG* S315T mutation and the -c15t *inh*A promoter mutation [8]. Next, for isolates without either of these two mutations, we sequenced the *inhA* promoter region and the entire *fur*A-*kat*G locus to identify other mutations [8]. The resulting mutations were sorted into four previously described groupings [8]: Group 1: isolates with a *katG* mutation other than S315T, Group 2: isolates with a *katG* S315T mutation, Group 3: isolates with a *inhA-15;c-t* promoter mutation, and Group 4: isolates with other or no identified mutations. We did not include mutations in *ahpC* because these do not appear to confer INH resistance [6].

### Definitions

Drug susceptibility was confirmed in all cases using the agar-proportion method. INH resistance was classified as either low-level or high-level when there was >1% growth of *M. tuberculosis* complex in the presence of 0.2 *ug*/mL or 1 *ug*/mL of INH, respectively. Clinical characteristics, including presenting symptoms, the presence or absence of cavitation on chest radiographs, and treatment outcomes were abstracted from the San Francisco Department of Public Health Tuberculosis Clinic electronic database and charts. Treatment was considered completed if all anti-tuberculosis chemotherapy had been administered and there was either microbiological confirmation of cure or an indication in the medical record that the patient had received an effective course of treatment. An adverse drug reaction was defined as any symptom or laboratory abnormality leading to interruption of ≥1 anti-tuberculosis medication. Treatment outcomes of interest included death on TB treatment, culture positivity 2 months after treatment initiation, and treatment failure or relapse. In accordance with ATS/CDC/IDSA guidelines, a patient was considered to have treatment failure if culture results remained positive after 4 months of treatment and to have had a relapse when a second episode of tuberculosis was diagnosed within one year after treatment completion.

### Statistical methods

We performed bivariate analysis using the chi-squared test or Fisher's exact test for categorical variables and Mann-Whitney rank-sum test for continuous variables. We performed logistic regression to measure the association between INH resistance-conferring mutation groups and lineage. We performed multivariate analysis using a Poisson regression model with robust error variance [13] to determine the association between INH resistance-conferring mutation group and high-level INH resistance. To account for potential confounding, we decided *a priori* to adjust for the following pre-specified variables: age, gender, foreign-born status, HIV serostatus, prior active TB treatment, and prior latent tuberculosis infection (LTBI) treatment. We evaluated trends in INH monoresistant TB cases by using Poisson regression, as previously described [14]. We performed all analyses in Stata 10.0 (Stata Corporation, College Station, TX).

## Results

### Study population

Of 137 TB cases with INH monoresistance identified during the study period, 36 were excluded from the analysis (25 isolates were not available, 2 isolates did not have sufficient DNA to analyze, 6 isolates had unknown INH resistance level (low vs. high), and 3 isolates were clustered). Complete demographic and clinical characteristics of the study population are shown in Table 1. Of 101 cases included in the analysis, 90 (89%) were pulmonary and 48 (47%) were sputum smear-positive. The median age of the study population was 43 years, 60% were male, 88% were foreign born, and 5% were HIV co-infected. In addition, 20 (20%) had prior treatment for active TB, and 19 (19%) had prior treatment for LTBI. High-level INH resistance was present in 63 (62%) cases. Demographic and clinical characteristics were similar between cases with high and low-level INH resistance, and between cases that were and were not included in the analysis (p>0.05 for all comparisons).

**Table 1:** Demographic and Clinical Characteristics.

Characteristic	N (%)
Male	61 (60)
Age, median years (IQR)	43 (29–65) years
Ethnicity	
Non-Hispanic white	7 (7)
Non-Hispanic black	4 (4)
Hispanic	10 (10)
Asian/Pacific Islander	79 (78)
Native American	1 (1)
Foreign-born	89 (88)
Homeless or single room occupancy hotel resident	9 (9)
History of drug abuse	8 (8)
History of alcohol abuse	12 (12)
HIV-infected	5 (5)
Prior active tuberculosis treatment	20 (20)
Prior latent tuberculosis treatment	19 (19)
Pulmonary tuberculosis	90 (89)
Positive AFB smear test	48 (47)
Fever	28 (28)
Night sweats	26 (26)
Weight-loss	33 (33)
Cough	50 (50)
Hemoptysis	16 (16)
Cavitary chest radiograph	16 (16)
High-level isoniazid resistance	63 (62)

Abbreviations:

AFB: Acid Fast Bacilli.

IQR: Interquartile Range.

### Drug-resistance alleles

**Figure 1:**
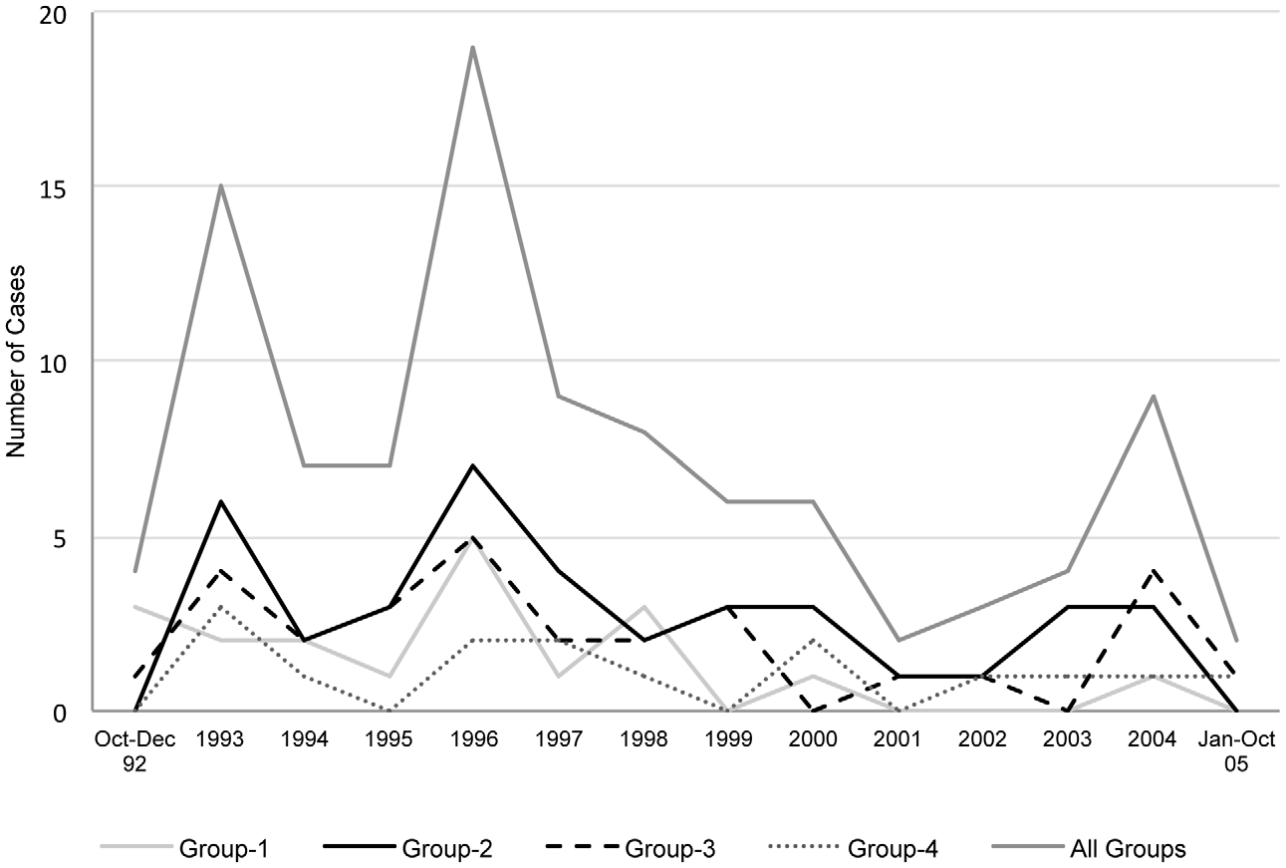
Isoniazid (INH) resistance-conferring mutations from 1992–2005 in San Francisco. Each line represents the number of cases in each INH resistance mutation group over time. The proportion of cases caused by isolates with group 1 mutations decreased significantly over the first four years of the study (p = 0.005), but there was no significant trend thereafter.

A *katG* mutation other than S315T was found in 19 isolates (Group 1); 38 isolates had the *katG* S315T mutation (Group 2), 29 isolates had a *inhA-15;c-t* promoter mutation (Group 3), and 15 isolates had other (8) or no identified (7) mutations (Group 4). No isolates had more than one mutation. There was no significant change in the proportion of cases caused by isolates in Groups 2 (p = 0.16), 3 (p = 0.19), and 4 (p = 0.68) during the 13-year study period (Figure 1). The proportion of isolates with Group 1 mutations decreased significantly during the first four years of the study (p = 0.005), but there was no significant change thereafter.

### Lineage

Of the 101 strains of *M. tuberculosis*, 17 (17%) belonged to the East-Asian lineage, 44 (44%) to the Euro-American lineage, 36 (36%) to the Indo-Oceanic lineage, and 4 (4%) to other lineages. Clinical presentation was not significantly different among these lineages (Table S1). A high proportion of cases of East-Asian lineage had *katG* mutation other than S315T (Group 1) (35.3%), and a high proportion of cases of Euro-American lineage had *katG* S315T mutations (Group 2) (40.9%). However, there were no significant associations between specific INH resistance-conferring mutations and genetic lineage (Table 2).

**Table 2:** Association between Three *M. tuberculosis* Strain Lineages and Isoniazid-Resistance Conferring Mutations.

	East-Asian Lineage	Euro-American	Indo-Oceanic	Other lineages	Odds Ratio	*P*-value
Mutation, n (%)	(n = 17)	(n = 44)	(n = 36)	(n = 4)	(95% CI)^a^	
***(1) katG*** ** other than S315T**	6 (35.3)	8 (18.2)	5 (13.9)	0 (0)	2.98 (0.94–9.48)	0.064
**(2) ** ***katG*** ** S315T**	4 (23.5)	18 (40.9)	14 (38.9)	2 (50.0)	1.28 (0.57–2.88)	0.55
***(3) inhA-15;c-t*** ** promoter**	4 (23.5)	9 (20.5)	14 (38.9)	2 (50.0)	2.12 (0.88–5.14)	0.096
**(4) Other or none identified**	3 (17.6)	9 (20.5)	3 (8.3)	0 (0)	2.19 (0.71–6.69)	0.171

a For each lineage, logistic regression was used to compare the proportion of the most frequent mutation to its proportion in the other lineages combined.

### Bivariate and Multivariate Analyses

In bivariate analysis (Table 3), there was no significant association between INH resistance- conferring mutation group and clinical presentation of TB (including cough, hemoptysis, fever, night sweats, weight-loss, and cavitary chest radiograph; p>0.05 for all comparisons). Demographic characteristics were similar across all INH resistance-conferring mutation groups (p>0.05) (Table 3). There were only three adverse clinical outcomes – one relapse after successful treatment (Group 2 mutation), one treatment failure resulting in death (Group 3 mutation), and one death during treatment (Group 2 mutation). However, the proportion of isolates with high-level INH resistance varied among different mutation groups, with 63% of Group 1, 92% of Group 2, 24% of Group 3, and 60% of Group 4 isolates exhibiting high-level INH resistance. When compared to isolates with Group 1 mutations, isolates with Group 2 mutations were more likely to have high-level INH resistance (92% vs. 63%, p = 0.04) and isolates with Group 3 mutations were less likely to have high-level INH resistance (24% vs. 63%, p = 0.01). After adjustment for age, gender, foreign-born status, HIV, and prior active or latent TB treatment (Table 4), Group 2 mutations remained associated with high-level INH resistance (risk ratio [RR] 1.56, 95% confidence interval [CI] 1.07–2.27, P = 0.02), and Group 3 mutations remained inversely associated with high-level INH resistance (RR 0.43, 95% CI 0.21–0.89, P = 0.02).

**Table 3:** Clinical Presentation Characteristics of Isoniazid (INH) Mono-Resistant Tuberculosis Cases by INH Resistance Group.

	INH Resistance Group (%)				
	Group 1	Group 2	Group 3	Group 4	*P-value*
Characteristic, n (%)	(n = 19)	(n = 38)	(n = 29)	(n = 15)	
**Fever**	9 (47)	9 (23)	7 (24)	3 (20)	*0.62*
**Night Sweats**	10 (52)	8 (21)	6 (21)	2 (13)	*0.17*
**Weight-loss**	8 (17)	13 (34)	8 (28)	4 (27)	*0.97*
**Cough**	13 (68)	18 (47)	13 (44)	6 (40)	*0.83*
**Hemoptysis**	6 (32)	7 (18)	3 (10)	0 (0)	*0.16*
**Cavitary Chest Radiograph**	4 (21)	7 (18)	3 (10)	2 (13)	*0.80*

**Table 4:** Association Between Isoniazid (INH) Resistance-conferring Mutations and High-Level INH Resistance.

Mutation Group	Total Cases	High-Level INH Resistance	Association with High-Level INH Resistance	*P*-value
		n (%)	Adjusted Risk Ratio (95% CI)^a^	
***(1) katG*** ** other than S315T**	19	12 (63)	reference	
**(2) ** ***katG*** ** S315T**	38	35 (92)	1.56 (1.07–2.27)	0.02
***(3) inhA-15;c-t*** ** promoter**	29	7 (24)	0.43 (0.21–0.89)	0.02
**(4) Other or none identified**	15	9 (60)	1.01 (0.62–1.64)	0.98

a Poisson regression with robust error variance was used to measure association between INH resistance-conferring mutation group and high-level INH resistance.

## Discussion

This study describes a large cohort of INH monoresistant TB cases, and is the only study of which we are aware to correlate specific INH resistance-conferring mutations with the clinical presentation and outcome of TB. We found that INH resistance-conferring mutations were associated with differing levels of phenotypic INH resistance. However, specific mutations did not impact the clinical presentation of TB, and there were few adverse outcomes across all mutation groups.

Our data are consistent with previous studies demonstrating an association between the *katG* S315T mutation and high-level INH resistance. *katG* encodes a mycobacterial catalase-peroxidase that also activates INH from its prodrug state, likely to an INH-nicotinamide adenine dinucleotide (NADH) adduct [15]. Mutations of the native serine residue at *katG* position 315 can alter the enzyme's ability to bind INH. An enzymatic kinetic study demonstrated that the *katG* S315T mutation was among the least active mutations in producing the INH-NADH adduct among 23 *katG* mutants tested [16]. Thus, MTB strains with the *katG* S315T mutations may activate less INH than other *katG* mutants, and therefore survive under higher concentrations of INH. However, our data suggest specific INH resistance-conferring mutations do not impact other clinical manifestations of TB.

Other studies have described an association between INH resistance-conferring mutation and genetic lineage, particularly between the *katG* mutation other than S315T and the East-Asian lineage, and between the *inhA-15;c-t* promoter mutation and Indo-Oceanic lineage [8]. While our study suggested an association between the *katG* mutation other than S315T and the East-Asian lineage, these results did not reach statistical significance.

There are several potential reasons for the lack of an association between INH resistance mutation group and clinical presentation of TB. First, it is possible that strains harboring the *katG* S315T mutation are more resistant to INH and more easily transmissible, but are no more virulent in humans than strains harboring other INH resistance-conferring mutations. This contrasts with murine studies showing increased virulence of strains with *katG* S315T mutation [17]. Second, host factors may be more important than pathogen-related factors in determining clinical manifestations of disease. For example, HIV infection and other immunocompromising conditions are associated with more severe disease and worse clinical outcomes [18] [19] [20]. Lastly, although our data are from one of the largest population-based series of INH monoresistance cases, our study may have been underpowered to detect a difference in clinical outcomes. The vast majority of patients with INH monoresistant TB had satisfactory treatment outcomes in our setting. Similar studies in either larger populations or populations with higher morbidity or mortality could reveal subtle differences in outcomes between TB cases resulting from different INH resistance-conferring mutations.

In conclusion, in our population-based sample of INH monoresistant TB cases, we confirmed the previously reported association between the *katG* S315T mutation and high-level INH resistance, and an association between *inhA* promoter mutations and low-level INH resistance. No mutation was associated with differing clinical manifestations of TB, and adverse outcomes were uncommon across all mutation groups. Our data suggest that INH resistance-conferring mutations may not impact the clinical presentation of *M. tuberculosis* and that routine genetic testing of INH monoresistant isolates is unlikely to have diagnostic or prognostic value. However, further studies are needed to determine whether specific INH resistance-conferring mutations impact clinical presentation or outcomes in the setting of HIV-co infection or multiple drug resistance.

## Supporting Information

Table S1 **Clinical Presentation of Isoniazid (INH) Monoresistant Tuberculosis Cases by Lineage.**
(DOC)
