# Supplementary material for: Impact of Isoniazid Resistance-Conferring Mutations on the Clinical Presentation of Isoniazid Monoresistant Tuberculosis
Source: PLoS One. 2012 May 23;7(5):e37956. doi: 10.1371/journal.pone.0037956 (PMC3359338; doi:10.1371/journal.pone.0037956)
Supplement: Table S1 — Clinical Presentation of Isoniazid (INH) Monoresistant Tuberculosis Cases by Lineage. (DOC) [file pone.0037956.s001.doc]

| **Supplementary Table S1: Clinical Presentation of Isoniazid (INH) Monoresistant Tuberculosis Cases by Lineage** | | | | | |
| --- | --- | --- | --- | --- | --- |
|  | **East-Asian Lineage** | **Euro-American** | **Indo-Oceanic** | **Other lineages** | ***P-*value** |
| **Characteristic, n (%)** | **(n=17)** | **(n=44)** | **(n=36)** | **(n=4)** |  |
| **Fever** | **6 (35)** | **13 (30)** | **9 (25)** | **0 (0)** | ***0.65*** |
| **Night Sweats** | **5 (29)** | **15 (34)** | **6 (17)** | **0 (0)** | ***0.23*** |
| **Weight-loss** | **3 (18)** | **15 (34)** | **14 (39)** | **1 (25)** | ***0.32*** |
| **Cough** | **11 (65)** | **20 (45)** | **17 (47)** | **2 (50)** | ***0.24*** |
| **Hemoptysis** | **5 (29)** | **5 (11)** | **5 (14)** | **1 (25)** | ***0.17*** |
| **Cavitary Chest Radiograph** | **0 (0)** | **9 (20)** | **7 (19)** | **0 (0)** | ***0.17*** |
